# Buckling Susceptibility of a K-File during the Initial Negotiations of Narrow and Curved Canals Using Different Manual Techniques

**DOI:** 10.3390/jcm11226874

**Published:** 2022-11-21

**Authors:** Filippo Santarcangelo, Vittorio Dibello, Laura Garcia Aguilar, Adriana Carmelita Colella, Andrea Ballini, Massimo Petruzzi, Vincenzo Solfrizzi, Francesco Panza

**Affiliations:** 1 Private Practice, 70100 Bari, Italy; 2 Department of Orofacial Pain and Dysfunction, Academic Centre for Dentistry Amsterdam (ACTA), University of Amsterdam and Vrije Universiteit Amsterdam, 1081 Amsterdam, The Netherlands; 3 Department of Civil, Environmental, Land, Building Engineering and Chemistry, Polytechnic University of Bari, 70125 Bari, Italy; 4 Department of Precision Medicine, University of Campania “Luigi Vanvitelli”, 80138 Naples, Italy; 5 Interdisciplinary Department of Medicine, Section of Dentistry, University of Bari Medical School, 70124 Bari, Italy; 6 Cesare Frugoni Internal and Geriatric Medicine and Memory Unit, University of Bari Aldo Moro, 70124 Bari, Italy; 7 Unit of Research Methodology and Data Sciences for Population Health, National Institute of Gastroenterology “Saverio de Bellis”, Research Hospital, 70013 Castellana Grotte, Italy

**Keywords:** endodontics, double-curved canal, pathfinding instruments, buckling, canal negotiation

## Abstract

(1) Background: One possible way to investigate the potential impact or susceptibility of buckling on different manual techniques is to measure compressive loads during canal negotiation. The higher their values, the easier and quicker the critical load level to buckling is reached, leading to possible instrument lateral deformation. The objective of the present study was to investigate the impacts of compressive loads on a small K-file manipulated with different techniques for canal negotiation in simulated narrow and curved canals. (2) Methods: The tooth model selected was a plastic double-curved premolar 23 mm long (DRSK Group AB, Kasernvagen 2, SE-281 35, Hassleholm, Sweden) with an extremely narrow canal lumen to mimic a very difficult anatomical scenario. An experienced endodontist performed the negotiation of 90 of these artificial teeth randomly assigned to 3 different groups of 30 blocks each, respectively, using 3 different techniques: Group A: watch winding/pull (WW) motion; Group B: balanced forces (BF) technique; Group C: envelope of motion (EOM). The measurement system was based on the use of a dynamometer, Instron, Ltd. (model 2525-818 2kN f.s.), linked to a data acquisition unit HBM MGC+ to test all the compression and tensile loads, including all the peaks. (3) Results: All data acquired were processed by the CATMAN AP HBM software. Multiple comparisons for the highest compressive loads estimated the mean difference between WW vs. BF techniques of 3.60 [95% confidence interval (CI): 2.85 to 4.35, *p* < 0.001], WW vs. EOM of −1.76 (95% CI: −2.11 to 1.40, *p* < 0.001), and BF vs. EOM −5.36 (95% CI: −6.04 to −4.67, *p* < 0.001). (4) Conclusions: In conclusion, among the tested manual motions, the BF technique (Group B) was the most susceptible to buckling with the highest compressive load. WW motion (Group A) and EOM (Group C) were less susceptible to buckling than the BF technique. Therefore, a pressure-free manipulation of manual files, such as WW motion or EOM, can help reduce the susceptibility to buckling during the negotiation of narrow-curved canals.

## 1. Introduction

Canal negotiation, by definition, is a process of exploration and catheterization of the canal from the orifice to the foramen. It is an essential step of root canal preparation since it provides understanding of the anatomy (including shape, size, length, and level of irregularity) and creates unimpeded access to the apical one-third.

The endodontic instruments, hand-operated, including the K-files or engine driven employed during this important step of canal preparation, are usually termed “path-finders”, and their performances are affected by several factors, such as pitch, taper, cross-section, heat tempering, metal type, tip geometry, and operator skills [1]. Therefore, the pathfinders are endodontic instruments used to explore and catheterize the canal. Despite the presence on the dental market of pathfinders with different geometries, designs, and materials, the negotiation of narrow-curved canals might be really hard and tiring, and sometimes frustrating even for skilled operators [2,3]. In particular, when anatomical limitations, such as narrowness and curvature are present, the negotiation becomes a very challenging procedure. In fact, iatrogenic complications, such as blockages, ledges, and file fractures might occur more frequently than in routine cases, thus preventing the endodontic instruments to reach the full working length (W.L.).

The negotiation of difficult canal anatomy requires pathfinders that should be ideally small to slide inside constricted spaces but also resistant to cyclic fatigue, torsional load, bending, and buckling, in order to safely and effectively progress up to the canal terminus even in the presence of the curvature [4]. By definition, buckling is the lateral deformation of an endodontic instrument when subjected to a compressive load in the direction of its axis [5]. There are very few clinical studies investigating the buckling phenomenon in endodontics. In fact, in endodontic files, the low resistance to buckling can produce elastic deformations that are able to prevent their advancements into the apical canal [6]. From the few available studies, it appears to be clear how this phenomenon affects both glide path and shaping files [7]. The elastic or plastic deformation of a pathfinder, a consequence of a low resistance to buckling, has a relevant impact on negotiation since it might hamper the apical progression of the instrument toward the canal terminus. Small stainless steel files are more resistant to buckling than small-sized nickel titanium rotary files; therefore, they should be used first in the process of exploration. Indeed, the resistance to buckling is a prerequisite permitting pathfinders to first obtain access and then progress into difficult canals [8,9]. Among the stainless steel ones, there are the C+ files (Maillefer/Dentsply, Ballaigues, Switzerland), which have a 0.04 mm/mm taper in the first 4 mm from the tip and 0.02 mm/mm along the rest of the shaft. Due to their tapers, they are more resistant to buckling than the traditional K-files, which have 0.02 mm/mm tapers along the entire shaft [9]. Even if C+ files are more resistant to buckling than K-files, they are too bulky and rigid to slide through a curved constricted space [1]. Traditional K-files are not only more resistant to buckling than nickel titanium ones but also more flexible than C+ files, making them suitable for the exploration of narrow-curved canals. They are a good balance between buckling resistance and flexibility; in addition, they are available in very small sizes (smaller than nickel titanium ones). For all these reasons, the great majority of clinicians select and use them first in clinical practice; we also selected them for our study to make it more realistic.

Directly evaluating the relationship between buckling and manual techniques for canal negotiation is a nearly impossible task because it requires observation of the instrument until its compressive displacement (buckling) happens during the negotiation; this cannot be achieved because each movement of the instrument is performed in a time frame of seconds and the process of exploration cannot be interrupted to visually control the shape of the instrument. In addition, to simulate the real procedure, the instrument should be moved inside an artificial canal without observing what happens inside it.

One possible way to investigate the potential impact (susceptibility) of buckling on different manual techniques is to measure compressive loads during canal negotiation. The higher their values, the easier and quicker the critical load levels to buckling (leading to instrument lateral deformation) are reached. The compressive load value thus represents an important parameter to understand how buckling can potentially affect the dynamics of a pathfinder. Tensile loads must also be considered when present since they are parameters of safety for manual techniques. Indeed, working on the outstroke physically prevents buckling from happening. From a clinical viewpoint, it brings a reduced risk of iatrogenic complications, such as ledging and blockage. The objective of the present study was to investigate the buckling susceptibility of a conventional K-file during the negotiation of narrow-curved canals using different manual negotiation techniques. We choose K-files because they are the pathfinders with the smallest diameter and taper, are usually used as the first instruments in difficult (as well as curved) canals, since “physically” they have a higher chance of reaching the root canal end.

## 2. Materials and Methods

There are many ways of handling endodontic instruments, but in the present study, three particular techniques of canal negotiation were assessed.

### 2.1. Watch Winding Motion

This is one of the most commonly used techniques and it enables a file to advance with small amplitude rotations, right (30 to 60 degrees) and left (30 to 60 degrees), as the instrument is pushed forward into the canal [10].

### 2.2. Balanced Forces Technique

The file is positioned inside the canal using a clockwise rotation (no more than 180 degrees) and light, apically directed pressure. A counterclockwise rotation (120 degrees or greater) with simultaneous downward pressure directly proportional to the file size enables the file to cut dentin. Finally, one to two outward-pull clockwise rotations without pressure promote debris removal. Usually, the desired length is reached after several clockwise placements and counterclockwise cutting rotations [11].

### 2.3. Envelope of Motion

Envelope of motion (EOM) is an engineering term that describes the removal of restrictive dentin as the pre-curved instrument is removed from the canal itself in a clockwise direction. The technique consists of pre-curving a file, sliding it up to the first light resistance, then making a rotation with simultaneous removal of the instrument. During each cycle of the envelope of motion, the instrument cuts the dentin laterally, allowing itself to get deeper until the working length is reached [12].

**Figure 1:**
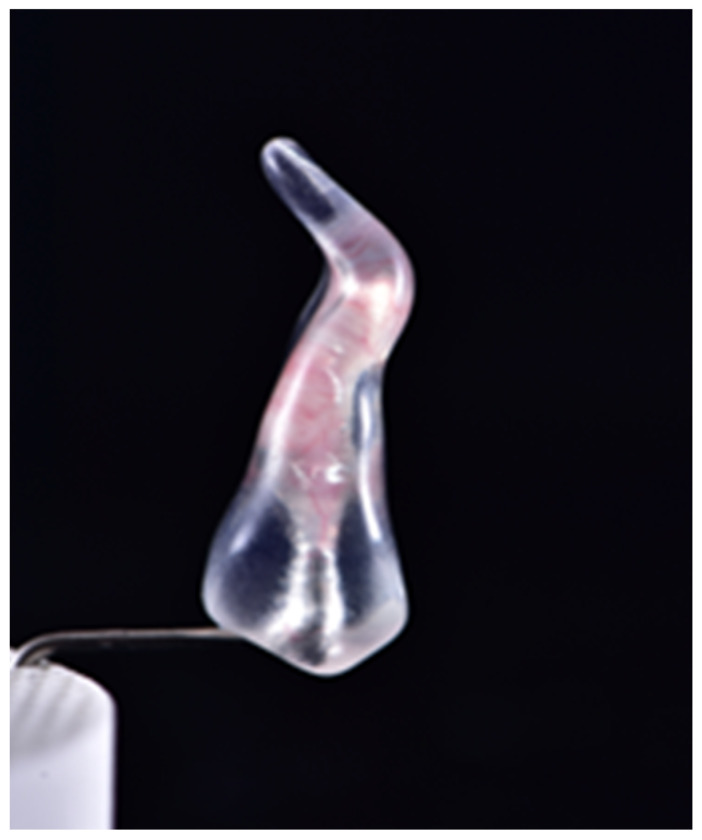
The selected double-curved tooth model.

The vertical tooth model (Figure 1) selected for the present study is a double-curved premolar that is 23 mm long (DRSK Group AB, Kasernvagen 2, SE-281 35, Hassleholm, Sweden), with an extremely narrow canal lumen and silicone content, to mimic a very difficult anatomical scenario.

The selected manual instrument was a 25 mm long K-file, (Maillefer/Dentsply, Ballaigues, Switzerland), a stainless steel instrument with a constant taper of 0.02 mm/mm since it is the most commonly employed by clinicians due to its multiplicity of uses (it can be used with filing and reaming movements and with all of the above-mentioned techniques). The size of the K-file was 0.10 mm; therefore, it was slightly bigger than the canal lumen of the artificial tooth (0.8 mm). It makes the negotiation technically more difficult and replicates the clinical situation of a severely narrow and curved canal.

**Figure 2:**
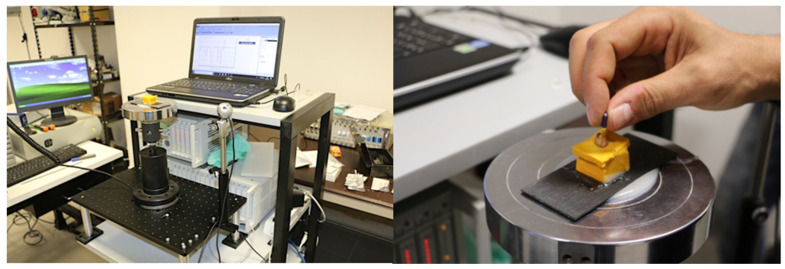
The testing machine. Full instrument equipment (**left** view) and K-file positioning (**right** view).

In this kind of situation, even when the smallest scouting file (0.6 K-file) is used, reaching the canal terminus remains very difficult. All tests were carried out at the Polytechnic of Bari in the Official Laboratory for Material Testing “Michele Salvati” located in the Department of Civil Engineering and Architectural Sciences (DICAR) with the support and supervision of an engineer (A.C.). The measurement system is based on the use of a dynamometer, INSTRON, Ltd. (model 2525-818 2kN f.s.- Instrom Europe, Bucks, UK), linked to a data acquisition unit HBM MGC+. All data acquired were then processed by the CATMAN AP HBM software (Figure 2).

The load cell was calibrated to 0.1 N. Considering that high accuracy is necessary to study the load generated by the use of a K-file during the root canal negotiation, the use of a load cell with an accuracy of 10 gr appeared to be appropriate. Each tooth model, previously filled with sodium hypochlorite, was mounted on a stable customized silicon support and the whole complex (tooth model and support base) was placed on the load cell. An experienced endodontist performed the negotiations of 90 of these artificial teeth (length = 23 mm) assigned to 3 different groups of 30 blocks each. The canals were, respectively, negotiated using the above-mentioned three techniques: Group A: watch winding (WW) motion; Group B: balanced forces (BF) technique; Group C: EOM.

After assuring that the apparatus was ready to record the loads and that the operator was ready to start the procedure, the recording of data could start. At this point, the K-file was introduced into the canal of the tooth model, allowing the load cell to record all compression and tensile loads, including the peaks. Compression loads were recorded when the K-file was inserted and pushed inside the canal root; on the contrary, tensile loads were measured when the K-file was pulled back. For each test, when the file reached the canal terminus, the recording of the loads was stopped. All recorded loads were saved with the CATMAN AP HBM software, Version v5.1.3.

### 2.4. Statistical Analysis

Descriptive statistical analyses of the three techniques WW, BF, and EOM by the highest and lowest compressive loads were summarized using means and standard deviations. Welch’s one-way analysis of variance (ANOVA) was used to compare variances across the means of the three techniques in the two compressive loads and Levene’s test was estimated to assess the equality of variances of this statistical analysis. Mean differences and 95% confidence interval(s) (CI) of multiple comparisons between the three techniques for the highest and lowest compressive loads were calculated by Dunnett's test. The *p*-value was set at 0.05. 

## 3. Results

The results for the WW motion (Group A), the BF technique (Group B), and the EOM (Group C) are depicted in Table 1.

**Figure 3:**
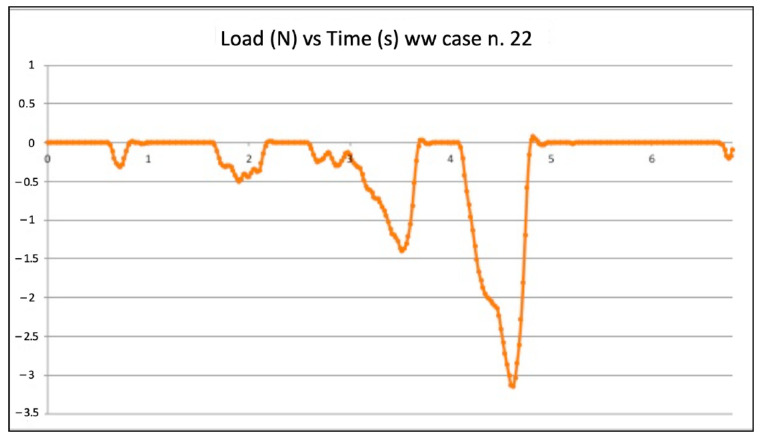
Graphic plot of the simulation on model n. 22 (failed watch winding motion negotiation).

**Figure 4:**
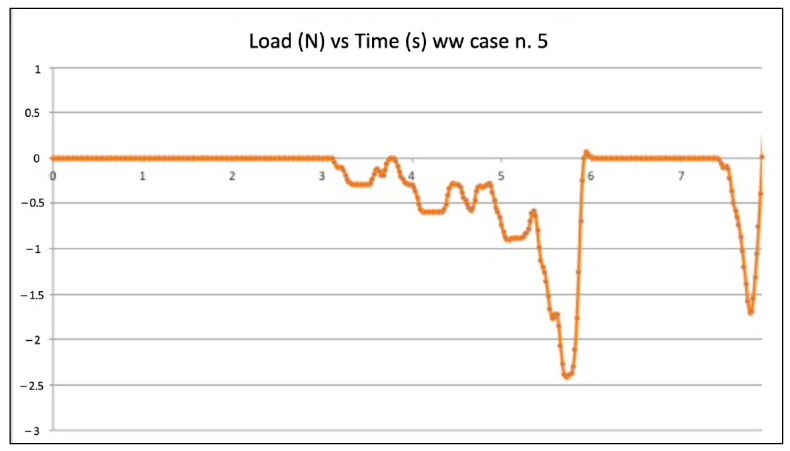
Graphic plot of the simulation on model n. 5 (successful watch winding motion negotiation).

Figure 3 and Figure 4 show the graphic plots of the simulation on model n. 22 (failed WW motion negotiation: the file could not reach W.L.) and model n. 5 (successful WW motion negotiation: the file reached W.L.). On the y-axis load (newton, N) on the x-axis time (seconds, s).

**Figure 5:**
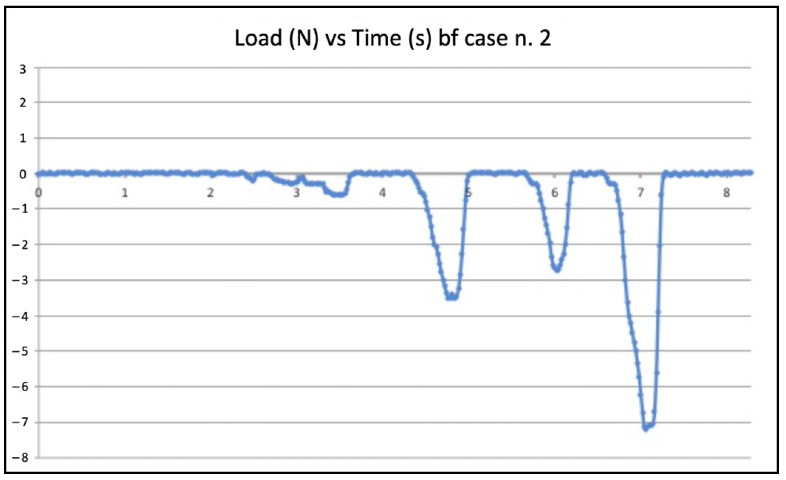
Graphic plot of the simulation on model n. 2 (failed balanced force technique negotiation).

**Figure 6:**
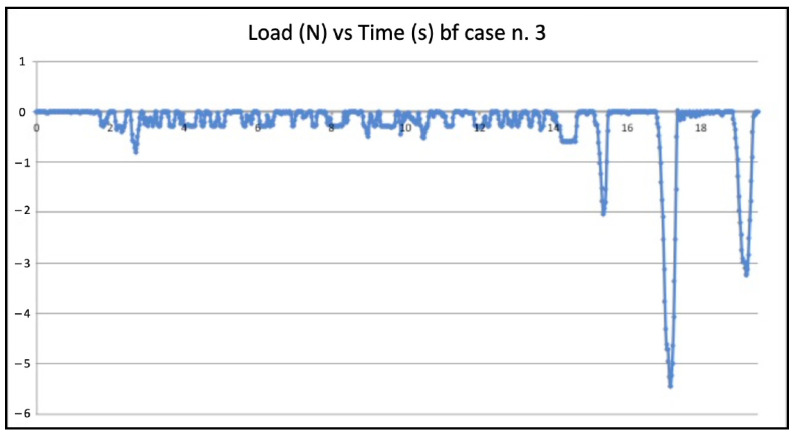
Graphic plot of the simulation on model n. 3 (successfully balanced force technique negotiation).

Five failed negotiations occurred in Group A (tooth n. 2, n. 6, n. 12, n. 20, n. 22). Figure 5 and Figure 6 show the graphic plots of the simulation on model n. 2 (failed BF negotiation: the file could not reach W.L.) and model n. 3 (successful BF negotiation: the file reached W.L.). On the y-axis load (newton, N) on the x-axis time (seconds, s).

**Figure 7:**
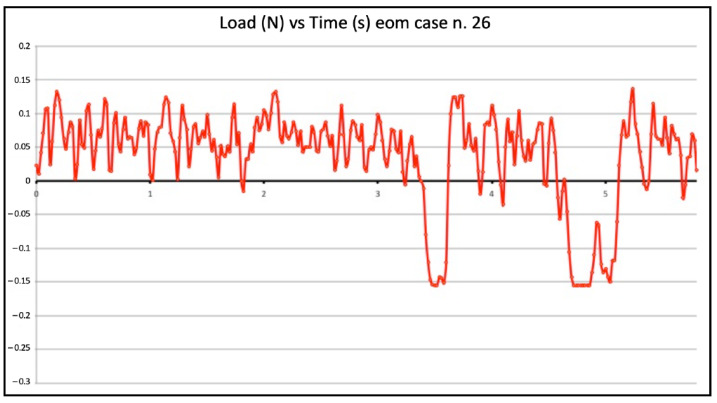
Graphic plot of the simulation on model n. 26 (successful envelope of motion negotiation).

**Figure 8:**
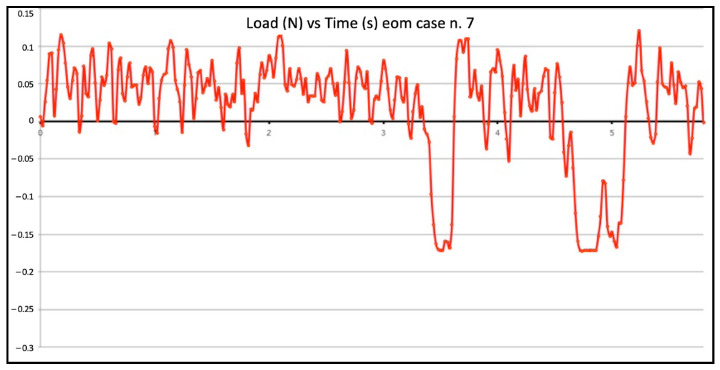
Graphic plot of the simulation on model n. 7 (successful envelope of motion negotiation).

**Figure 9:**
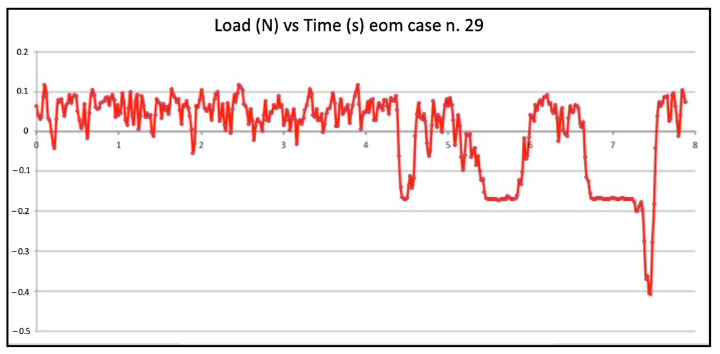
Graphic plot of the simulation on model n. 29 (failed envelope of motion negotiation).

Seven failed negotiations occurred in Group B (tooth n. 2, n. 5, n. 9, n. 14, n. 20, n. 22, n. 25). Figure 7 and Figure 8 show the graphic plots of the simulation on model n. 26 and n. 7 (successful EOM negotiations), while for Group C, Figure 9 shows the graphic plot of the simulation of model n. 29, the only failed case. On the y-axis load (newton, N), and on the x-axis time (seconds, S). For all groups, the deformed files were the ones involved in the failed negotiation, i.e., n. 5 for Group A, n. 7 for Group B, and n. 1 for Group C; we did not find any broken files.

Descriptive statistics of the three techniques WW, BF, and EOM by compressive loads are summarized in Table 2.

**Figure 10:**
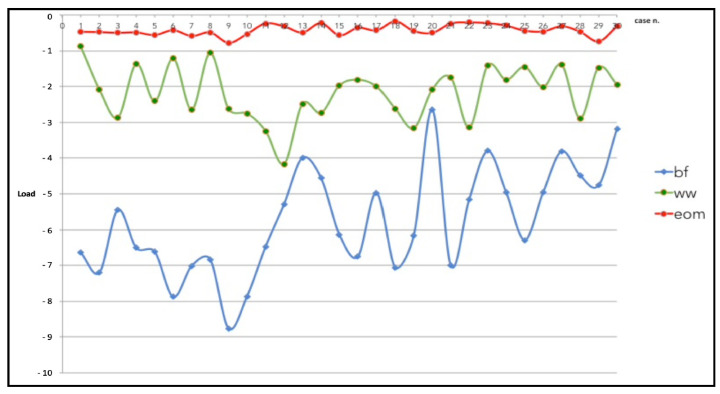
Comparison among the highest compressive loads of the balanced forces (BF) technique, watch winding (WW) motion, and the envelope of motion (EOM) in the thirty models.

Levene’s test was statistically significant, and we reject the hypothesis that the variance was homogeneous (*p* < 0.001 for the highest compressive load and *p* < 0.021 for the lowest compressive load). Consequently, because the homogeneity of variance assumption was used, the Welch statistic was 263.549, *p* < 0.001 for the highest compressive load and 98.878, *p* < 0.001 for the lowest compressive load). Multiple comparisons for the highest compressive loads estimated a mean difference between WW vs. BF techniques of 3.60 (95% CI: 2.85 to 4.35, *p* < 0.001), WW vs. EOM of −1.76 (95% CI: −2.11 to 1.40, *p* < 0.001), and BF vs. EOM −5.36 (95% CI: −6.04 to −4.67, *p* < 0.001). Figure 10 shows the graphic plot of the comparison among the highest compressive loads of the three groups. The BF technique showed the highest values of the load followed by the WW motion and, at last, the EOM.

**Figure 11:**
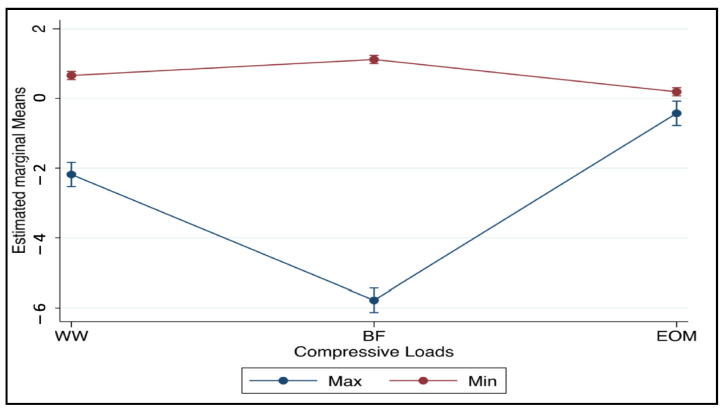
Estimated prediction means of the balanced forces (BF) technique, watch winding (WW) motion, and the envelope of motion (EOM) by the highest and lowest compressive loads with 95% confidence intervals.

Figure 11 shows the estimated prediction means of the three techniques WW, BF, and EOM by the highest and lowest compressive loads with 95% CI.

**Figure 12:**
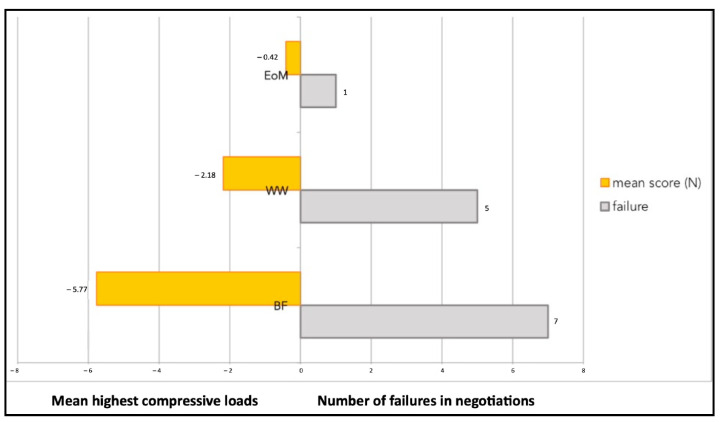
The mean highest compressive load values (newton, N) and the number of failures in negotiations of the balanced forces (BF) technique, watch winding (WW) motion, and the envelope of motion (EOM). On the y-axis, the different techniques used are presented.

Figure 12 shows the graphic plot of the highest compressive load values and percentages of success in negotiations of the three groups.

The higher the compressive load value of the technique, the lower the percentage of success in negotiation; therefore, the success was inversely proportional to the compressive load.

## 4. Discussions

In the present study, by investigating the impacts of compressive loads on a small K-file manipulated with different techniques (WW motion, BF technique, and EOM) for canal negotiation in simulated narrow and curved canals, the BF technique (Group B) was the most susceptible to buckling with the highest compressive load. WW motion (Group A) and EOM (Group C) were less susceptible to buckling than the BF technique.

Some limitations of the present study must be acknowledged. First of all, we did not perform a sample size calculation for the present pilot study without a control group. Although natural teeth represent the best option to evaluate the dynamics of a pathfinder inside the root canal system, their main limitation is due to differences in size, length, and curvature of the canal, leading to a very difficult standardization. In addition, the absence or the presence of the canal content (type and consistency) plays a role in complicating the standardization. In the present study, resin tooth models were employed to better standardize the experimental conditions even if they remain an artificial reproduction of the anatomy.

Buckling resistance has been investigated in a few studies using nickel titanium instruments for glide path preparation depending on the test mode (static vs. dynamic) [6] or by testing various single-file systems (TruNatomy, WaveOne gold, and XP-Endo Shaper) [7]. In the present study, in an attempt to replicate the real anatomical condition, a double-curved tooth model that was 23 mm long was employed. Apart from its first 3 coronal mm, where some taper is provided by the manufacturer, the canal consists of a non-tapered space with an 0.8 diameter filled with a very soft silicone foam to simulate the canal content. Regarding the shape of this model, there is a double curvature: the coronal curvature located 7 mm from the canal terminus has a 127° angle, while the apical one is located 4 mm from the canal terminus and has a 160° angle (data from the manufacturer). The size of the working file (0.10 mm) was selected in relation to the diameter of the artificial canal (0.8 mm) to simulate a difficult anatomical situation, which is typical of severely narrow canals whose orifice and lumen are small enough to hamper the progression of the smallest file (i.e., 0.6 mm K-file) for negotiation.

**Figure 13:**
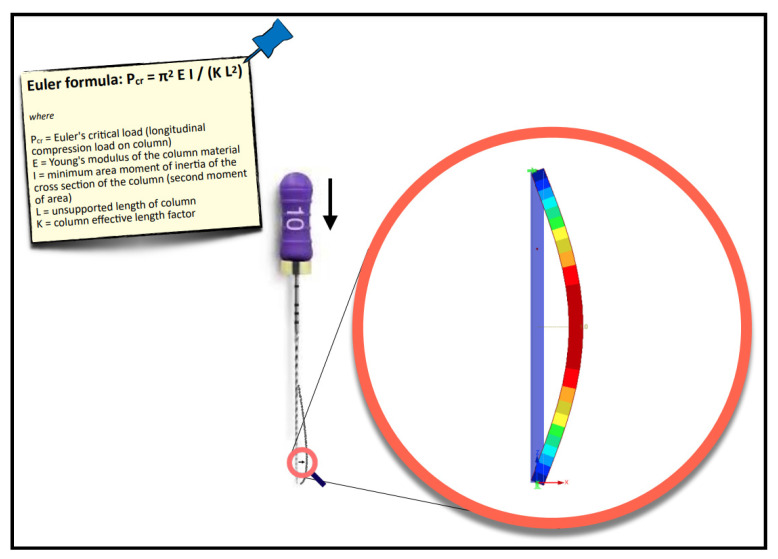
Schematic representation of the buckling of a K-file under compressive load (the Euler formula).

In clinical practice, file sizes smaller than 0.10 mm are usually necessary to start the management of constricted canals because they are the only ones able to get into narrow orifices [13]. On the other hand, their flexibility helps to follow the canal curvature. In this regard, the three main anatomical limitations in endodontics are canal curvature, narrowness, and length. Curvature and narrowness represent true obstacles (constraints, from a physical viewpoint) for the endodontic instrument; the more severe the obstacle to overcome, the bigger the friction hampering the file progression and the higher its possibility to buckle under the clinician’s fingers pressure [14]. To complicate matters, in canals already narrow and/or curved, the canal length also plays a role, especially because long canals are usually managed with long or very long (25 or 31 mm long) manual files. According to physics, in particular, the Euler formula, *P* = *π^2^ E I /K L*^2^, the instrument behaves, such as a slender column (Figure 13). 

The longer it is, the bigger its lateral deformation will be when it is apically advanced [15]. As a consequence, and in order to reduce the impact of buckling in long canals, the use of shorter files (21 mm) is warmly advised to start the negotiation [16]. They will basically work in the coronal and middle third, reducing the risk of early procedural complications related to the file’s impossibility of properly moving downward as a result of buckling [17].

Of course, longer files will be needed later to obtain the full working length but they will work in a safer situation where part of the work is already carried out by short and more effective files, physically less affected by buckling. The use of shorter K-files is also advised in canals having normal lengths but with a narrow lumen or a curvature; this brings the advantage of reduced buckling but also better tactile feedback experienced by the clinicians since the distance between their fingers and the file tip is less than in case of long hand files. It should be kept in mind that regardless of all efforts to reduce the impact of buckling, the small flexible files (06–08–10) normally involved in the negotiation of difficult anatomies are less resistant to buckling than the bigger or more tapered ones; the latter are virtually better solutions under a purely physical perspective. However, from a clinical viewpoint, increasing the size or the taper of a pathfinder dramatically hampers its insertion and progression in narrow-curved canals [1].

For the above-mentioned reasons, small files remain the most commonly used instruments for the negotiation of difficult canals and the ability of the clinician to properly handle them is making the difference. The compressive and tensile load values can be considered parameters of susceptibility to buckling and help to understand how buckling can potentially affect the dynamics of a pathfinder.

Among the tested manual motions, the most susceptible to buckling (highest compressive load) was the BF technique (Group B) because the entire work was made under compression loads and it is the only endodontic motion where the use of a higher force is allowed; of course, this force should match the file strength and size and be heavier for bigger files and lighter for the smaller ones. In particular, during the counterclockwise rotation, the force should be typically exerted to advance the file apically and this explains the findings of this study, showing the higher incidence of buckling for Group B (BF technique). From a clinical viewpoint, this high compressive load value adversely affected the percentage of success in negotiation, even if the inventor, Dr. Roane, advised this technique for curved anatomy because it promotes an apical progression of the file. Dr. Roane also claimed that small instruments, i.e., no. 8 and no. 10, can be crushed past calcifications and allow opening calcified canals to rapidly reduce, hence, the incidence of secondary blockages from loosened particles. 

The WW motion (Group A) was less susceptible to buckling than the BF technique (Group B); this can be explained by the minimal apical pressure that is exerted during the WW motion. However, even this minimal value of vertical force can be enough to buckle a small flexible file inside a constricted-curved canal, such as the model employed in the present study [17]; this also explains why the WW motion developed bigger compressive loads than EOM. WW motion is a “minimal-pressure” technique but if this pressure is too minimal, the K-file cannot be rotated back and forth and simultaneously advanced. In contrast, EOM is a “no-pressure technique” because the first step is to slide and glide until tactilely feeling the first very light resistance; in this approach, it is the canal that guides the instrument and not the operator. The entire work is performed on the outstroke with a simultaneous clockwise rotation and removal of the pre-curved instrument. Dentin is laterally carved, and after each outstroke cycle, the instrument passively moves deeper. It should be underlined that “working on the out-stroke” physically prevents buckling from happening. EOM is a technique that predominantly develops tensile loads and this makes it less susceptible to buckling. The great majority of the time is spent working on the outstroke. On the graph, all of the K-file movements above the zero line were performed in a safe area with no susceptibility to buckling. Comparing the results from the different groups, it is obvious that potentially critical values of compressive loads play relevant roles in adversely affecting the K-file progression during canal negotiation. 

Inside each group (WW motion, BF technique, and EOM), there were failures. The values of compressive loads for each case of failure were examined to check if they were higher than in the successful cases of the same group. It is quite clear that those values from failed negotiations were not significantly different from the average value of the same technique. However, they were always slightly higher, indicating that in the failed cases, the instrument experienced some constraints during progression. As an example, the BF technique failed negotiation n. 2 (Figure 3) showed slightly higher values of compressive loads than in successful case n. 3 (Figure 4). Considerations are the same for the WW motion and EOM. It can be speculated that a small increase in the compressive load in difficult anatomies is able to create the critical load, finally leading to a file buckling; but it is nearly impossible for a clinician to calibrate the exerted pressure for such minimal values of loads. Since the failure values were not significantly higher than success values, it remains to explain why similar compressive loads could lead to failure in a few cases and success in the great majority. The involved instrument and initial anatomy being the same, this result can be understood when focusing on the canal content.

The soft material simulating the canal content might be pushed and compacted apically along with plastic debris, creating friction, and hampering the progression of the K-file. The poor penetration of the irrigant during this stage might also play a relevant role in promoting this complication; a similar phenomenon might happen in reality even if the operator is skillful, providing evidence of how challenging the negotiation of constricted curved canals is. It can also be pointed out that even if the operator is prudent and light-handed in difficult anatomies, it is extremely easy to reach critical values of compressive loads. It can also be speculated that clinicians usually work the instruments with values of compressive loads that are potentially critical and, therefore, are able to create buckling. Despite this, in normal anatomical conditions, there is an absence of relevant obstacles (absence of constraints and relevant friction); this enables the instrument to be easily “dropped” inside the canal space and then reach the working length. On the other hand, when obstacles are found (narrow areas, curves, collagenous tissue) they act as physical constraints, and even if the clinician exerts the same force s/he usually exerts (a “routine” force), the friction may become relevant, the force becomes critical, and the instrument stops and buckles.

Through root canal shaping, endodontic files are subjected to various forces, such as shear, torsion, flexion, traction, and apical pressure [18,19]. Since flexural fracture was the primary fracture mode for instruments with larger tapers, a smooth glide path should be created when shaping the canal [18,19]. Hampering the progression, the fracture is just the final step if, despite the deformation, the operator keeps pushing or moving in the root canal. However, the fracture is less frequent than simple deformation and the inability to progress. As it is difficult to establish the instrument failure in clinical practice, a check on the K-file instrument is suggested prior to use. It is important to avoid overuse and to dispose of the instruments on a regular basis.

In the present study, in all tests (regardless of the technique, success, or failure), there is an evident relationship between anatomical impediment and susceptibility to buckling: in the final part of the negotiation, when the file approaches canal curvatures, higher values of compressive loads can be seen, and in particular, at least two peaks of compressive loads. All the findings from the present study suggest that in anatomically complex cases (constricted-curved canals), an extremely light manipulation promotes the instrument progression up to the canal terminus despite adverse physical conditions.

## 5. Conclusions

By investigating the impact of compressive loads on a small K-file manipulated with different techniques for canal negotiation in simulated narrow and curved canals, within the limitations of the present study, it can be concluded that a pressure-free manipulation of hand files, such as WW motion or EOM, may be helpful during the negotiation of narrow-curved canals, reducing the susceptibility to buckling.

## Figures and Tables

**Table 1:** Watch winding (WW) motion, balanced force (BF), and envelope of motion (EOM) of the highest and lowest compressive loads.

Watch Winding (WW)			Balanced Force (BF)			Envelope of Motion (EOM)		
Model n.	Highest Comprehensive Load (N)	Lowest Comprehensive Load (N)	Model n.	Highest Comprehensive Load (N)	Lowest Comprehensive Load (N)	Model n.	Highest Comprehensive Load (N)	Lowest Comprehensive Load (N)
	Mean score = −2.18 N	Mean score = 0.66 N		Mean score = −5.78 N	Mean score = 1.12 N		Mean score = −0.42 N	Mean score = 0.19
1	−0.88	0.02	1	−6.63	0.00	1	−0.47	0.05
2	−2.08	0.70	2	−7.20	1.66	2	−0.48	0.26
3	−2.87	0.94	3	−5.45	1.10	3	−0.50	0.10
4	−1.37	2.29	4	−6.51	1.25	4	−0.49	0.11
5	−2.41	0.57	5	−6.62	1.25	5	−0.56	0.33
6	−1.21	0.60	6	−7.88	1.29	6	−0.43	0.27
7	−2.65	0.61	7	−7.01	1.41	7	−0.59	0.39
8	−1.06	0.84	8	−6.83	1.10	8	−0.39	0.26
9	−2.63	0.46	9	−8.76	1.42	9	−0.78	0.34
10	−2.75	0.82	10	−7.86	1.25	10	−0.53	0.06
11	−3.24	0.87	11	−6.48	0.84	11	−0.24	0.17
12	−4.16	0.74	12	−5.29	1.39	12	−0.32	0.22
13	−2.48	0.99	13	−3.99	0.84	13	−0.49	0.04
14	−2.74	0.57	14	−4.55	1.05	14	−0.23	0.07
15	−1.97	0.58	15	−6.14	1.11	15	−0.57	0.33
16	−1.81	0.41	16	−6.75	0.78	16	−0.36	0.11
17	−2.00	0.57	17	−4.97	0.97	17	−0.43	0.45
18	−2.62	0.79	18	−7.06	1.40	18	−0.18	0.12
19	−3.16	1.00	19	−6.16	1.03	19	−0.45	0.14
20	−2.08	0.70	20	−2.64	0.11	20	−0.50	0.14
21	−1.75	0.55	21	−7.00	1.60	21	−0.24	0.16
22	−3.15	0.51	22	−5.15	1.03	22	−0.21	0.09
23	−1.41	0.54	23	−3.80	1.15	23	−0.23	0.10
24	−1.81	0.05	24	−4.96	1.14	24	−0.30	0.32
25	−1.45	0.79	25	−6.29	1.15	25	−0.44	0.15
26	−2.01	0.10	26	−4.96	1.42	26	−0.46	0.43
27	−1.39	0.04	27	−3.81	1.38	27	−0.32	0.14
28	−2.89	0.71	28	−4.84	1.18	28	−0.47	0.13
29	−1.47	0.57	29	−4.67	1.41	29	−0.74	0.18
30	−1.94	0.88	30	−3.18	0.85	30	−0.32	0.09

**Table 2:** Descriptive statistical analysis of the watch winding motion, balanced forces technique, and envelope of motion, each on 30 tooth models, by the highest and lowest compressive loads (N).

		Mean	Standard Deviation	Standard Error	95% Confidence Intervals for the Mean			Min.	Max.
**Highest compressive load**	Watch winding motion	−2.18	0.76	0.14	−2.47	−1.90	−4.16	−0.88	
	Balanced forces technique	−5.78	1.48	0.27	−6.33	−5.23	−8.76	−2.64	
	Envelope of motion	−0.42	0.15	0.03	−0.48	−0.37	−0.78	−0.18	
**Lowest** **compressive load**	Watch winding motion	0.66	0.41	0.07	0.51		0.81	0.02	2.29
	Balanced forces technique	1.12	0.36	0.07	0.98		1.25	0.00	2.66
	Envelope of motion	0.19	1.12	0.02	0.15		0.24	0.04	0.45

## Data Availability

Not applicable.
